# Supplementary material for: Dopamine Modulates the Processing of Food Odour in the Ventral Striatum
Source: Biomedicines. 2022 May 12;10(5):1126. doi: 10.3390/biomedicines10051126 (PMC9138215; doi:10.3390/biomedicines10051126)
Supplement: Supplementary file 1 [file biomedicines-10-01126-s001.zip › biomedicines-1685294-supplementary.pdf]

Supplementary Materials:

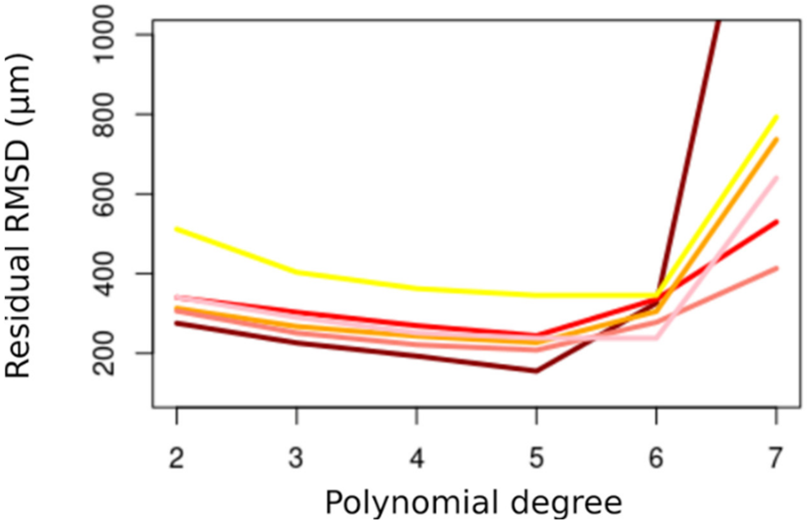

Figure S1. Residual root-mean square distance (RMSD) between anatomical landmark structures was computed after polynomial warping with polynomial degrees ranging from 2 to 7. A degree of 5 was chosen for spatial normalisation as it minimized the RMSD for all of the six coronal planes (curves are displayed in *dark-red*, *red*, *orange*, *salmon*, *pink* and *yellow* for reference coronal planes 1 to 6, respectively).
